# Supplementary material for: Widely Targeted Metabolomic Profiling Combined with Transcriptome Analysis Provides New Insights into Lipid Biosynthesis in Seed Kernels of Pinus koraiensis
Source: Int J Mol Sci. 2023 Aug 17;24(16):12887. doi: 10.3390/ijms241612887 (PMC10454069; doi:10.3390/ijms241612887)
Supplement: Supplementary file 1 [file ijms-24-12887-s001.zip › Figure S.pdf]

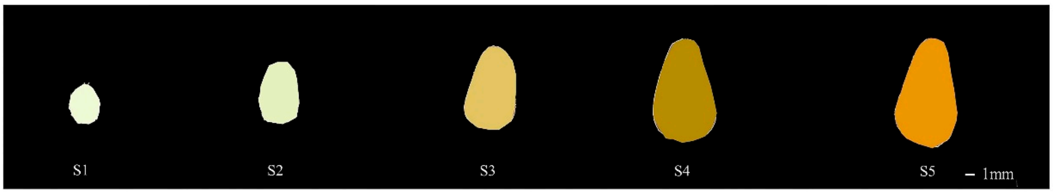

Figure S1. Phenotypic changes in seed kernels at five development stages, scale bar = 1 mm.

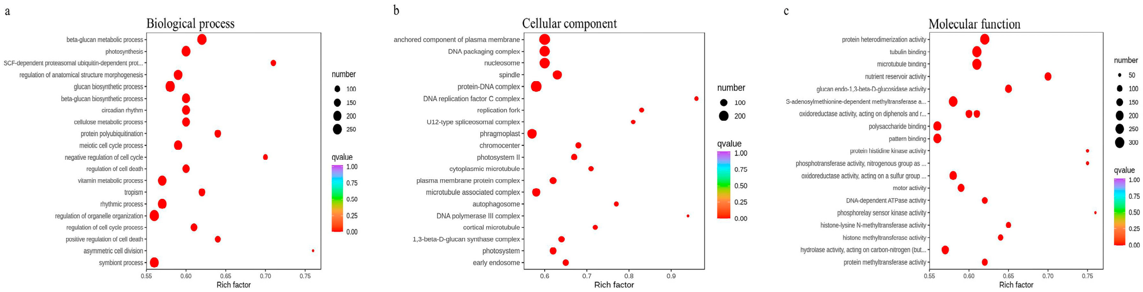

Figure S2. Gene Ontology enrichment analysis of differentially expressed genes during development of *Pinus koraiensis* seed kernels. (a) Biologic process enrichment analysis. (b) Cellular component enrichment analysis. (c) Molecular function enrichment analysis.

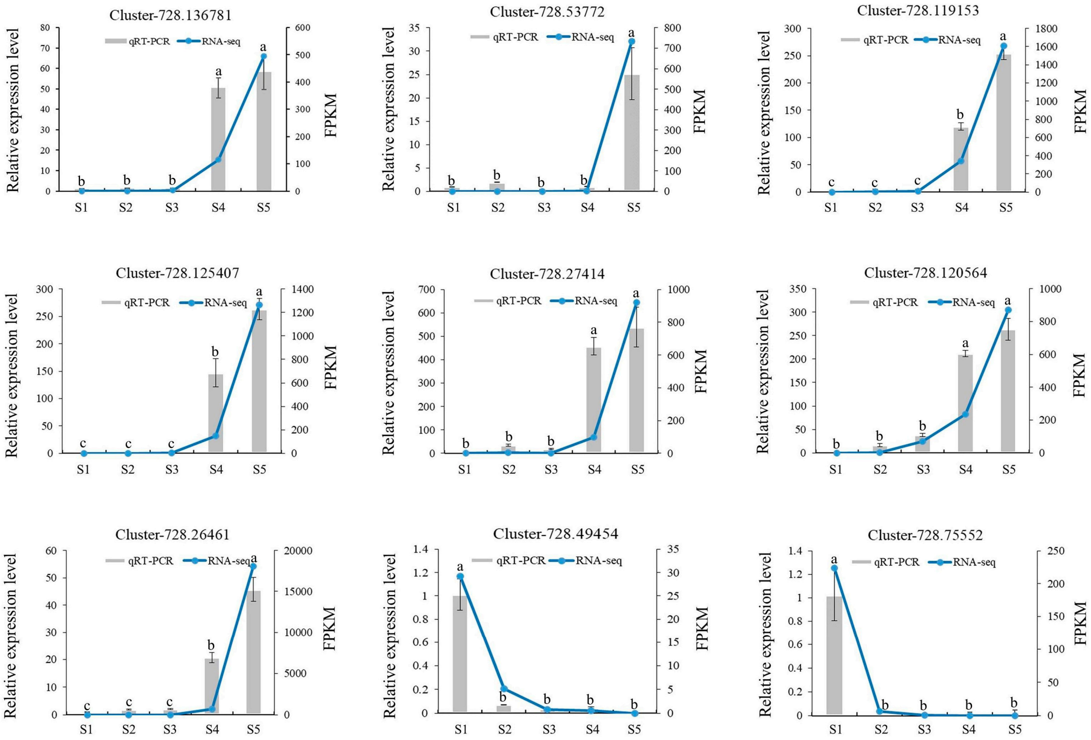

Figure S3. RT-qPCR verification of expression level of 9 selected candidate genes in transcriptome data. Samples at days of the year 130, 160, 190, 220, and 240 as test materials and named them as S1-S5. The differences analysis was conducted using the IBM SPSS Statistics v26.0 software with the Student-Newman-Keuls multiple range test; Error bars represent the SD of the means at n=3, bars with different lowercase letters are significantly different ($P<0.05$). The 18S gene was utilized as a reference gene, and S1 stage was used as a reference sample to calculate the 2-delta-delta Ct value.
